# ProtVar: mapping and contextualizing human missense variation

**DOI:** 10.1093/nar/gkae413

**Published:** 2024-05-20

**Authors:** James D Stephenson, Prabhat Totoo, David F Burke, Jürgen Jänes, Pedro Beltrao, Maria J Martin

**Affiliations:** EMBL-EBI, Wellcome Genome Campus, Hinxton CB10 1SD, Cambridgeshire, UK; EMBL-EBI, Wellcome Genome Campus, Hinxton CB10 1SD, Cambridgeshire, UK; Kings College London, London WC2R 2LS, UK; Department of Biology, Institute of Molecular Systems Biology, ETH Zürich, Zürich, Switzerland; Department of Biology, Institute of Molecular Systems Biology, ETH Zürich, Zürich, Switzerland; Swiss Institute of Bioinformatics, Lausanne, Switzerland; EMBL-EBI, Wellcome Genome Campus, Hinxton CB10 1SD, Cambridgeshire, UK

## Abstract

Genomic variation can impact normal biological function in complex ways and so understanding variant effects requires a broad range of data to be coherently assimilated. Whilst the volume of human variant data and relevant annotations has increased, the corresponding increase in the breadth of participating fields, standards and versioning mean that moving between genomic, coding, protein and structure positions is increasingly complex. In turn this makes investigating variants in diverse formats and assimilating annotations from different resources challenging. ProtVar addresses these issues to facilitate the contextualization and interpretation of human missense variation with unparalleled flexibility and ease of accessibility for use by the broadest range of researchers. By precalculating all possible variants in the human proteome it offers near instantaneous mapping between all relevant data types. It also combines data and analyses from a plethora of resources to bring together genomic, protein sequence and function annotations as well as structural insights and predictions to better understand the likely effect of missense variation in humans. It is offered as an intuitive web server https://www.ebi.ac.uk/protvar where data can be explored and downloaded, and can be accessed programmatically via an API.

## Graphical Abstract

**Graphical Abstract:**
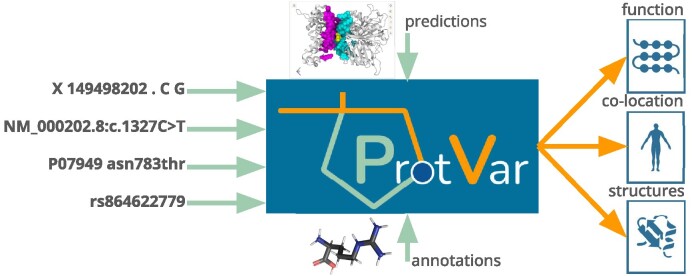

## Introduction

Mutagenesis underpins the genetic adaptation which is fundamental to evolution and life. Unravelling the connection between variations and the resultant phenotypic change in organisms is therefore fundamental to biological understanding. Specifically, variant consequence research has led to discoveries in host-pathogen interactions (1), drug resistance (2), identifying disease associations (3), disease susceptibility (4) and prognosis (5) as well as drug development (6). However, whilst the number of variants available via sequencing projects continues to rise, the majority are still classified as of ‘unknown significance’ (7) because their effects are reliant on many interacting considerations. Although annotations of function are also expanding, the position and environmental specificity of amino acid roles in proteins mean that this knowledge must be assimilated in as complete and meaningful a way as possible. The interpretation of the molecular effects of variants on proteins is still hindered by challenges in accurate positional mapping, data integration and standardization.

There are several excellent tools to help users to gather some of the information necessary to understand the potential consequences of genetic variation, each with their own specialities. For variant analyses at the genetic level, VEP (8) is a rich resource of in depth and highly trusted information. PhyreRisk (9) extends this offering a whole protein view to allow the exploration and mapping of genetic variants from genomic coordinates onto proteins using the VEP resource. Missense3D (10) focuses primarily on the interpretation of single missense variants based on user provided protein structures. Mutfunc (11) is a useful resource for investigating PTMs, stability changes and interactions. MisCast (12) has a wealth of protein level annotations for the 1330 human genes covered by the resource. Varsite (13) is another resource with wide ranging annotations but is no longer being developed. Decipher (14) is widely used by the clinical community to share genetic and phenotypic data. Other resources such as dbNFSP (15,16) VarCards and ANNOVAR (17) can also retrieve protein annotations. Each of the mentioned resources have specific uses and users should be encouraged to consider more than one resource for a complete understanding of the role of their variants.

Supplementary Table S1 shows a summarized comparison of variant analysis resources. Whilst this shows the cumulative depth in knowledge from these resources, it also highlights the limitations and specific use cases in each. We have developed ProtVar www.ebi.ac.uk/protvar to address these limitations and expand upon many of the most useful features of existing tools in a fast, updated, intuitive and flexible resource. We have integrated the most relevant data and features for the interpretation of missense variation. ProtVar is as user friendly as possible in order to be useful to the broadest range of researchers. The flexibility both in terms of input types (genomic/cDNA/protein/IDs) and formatting mean that users with mixed or messy data can both retrieve annotations and a standardized mapping without having to spend time formatting their data first. This means that users can retrieve protein and structure functional annotations directly from genomic inputs. It offers a broad range of curated, evidenced high throughput data annotations and predictions which are intuitively categorized to highlight key points but allow deeper investigation with links to complementary resources. ProtVar allows the community to quickly assess a small number of variants or an entire study and retrieve results via a download for further analysis or integrate data programmatically via the API. This website is free and open to all users and there is no login requirement.

## Materials and methods

### Input variant mapping

Four broad types of variant inputs can be processed by ProtVar, genomic, cDNA, protein and variant ID as shown in Table 1. Within each of these four broad categories several different formats are supported. ProtVar is as forgiving as possible in terms of white spaces and delimiters to reduce user preprocessing and save time.

**Table 1.** Variant input types which can be processed by ProtVar. Fields in brackets are optional

Input type	Format	Parameters	Examples
**Genomic**	VCF	chromosome, position, (id), (ref), (alt)	X 149498202. C G
	gnomAD/custom		1–55505447-C-T 14 89 993 420 A/G
	HGVSg	RefSeq, type, position, ref, alt	NC_000010.11:g.43118436A > C
**cDNA**	HGVSc	RefSeq, type, position, ref, alt (consequence)	NM_000202.8:c.1327C > T NM_020975.6(RET):c.3105G > A (p.Glu1035Glu)
**Protein**	UniProt accession and position	accession, position, (ref), (alt)	P22309 71 Gly Arg
	HGVSp	RefSeq, type, ref, position, alt	NP_001305738.1:p.Pro267Ser
**Variant ID**	dbSNP	Variant ID	rs864622779
	ClinVar		RCV001270034, VCV002573141
	COSMIC		COSV64777467, COSM1667583

**Figure 1.**
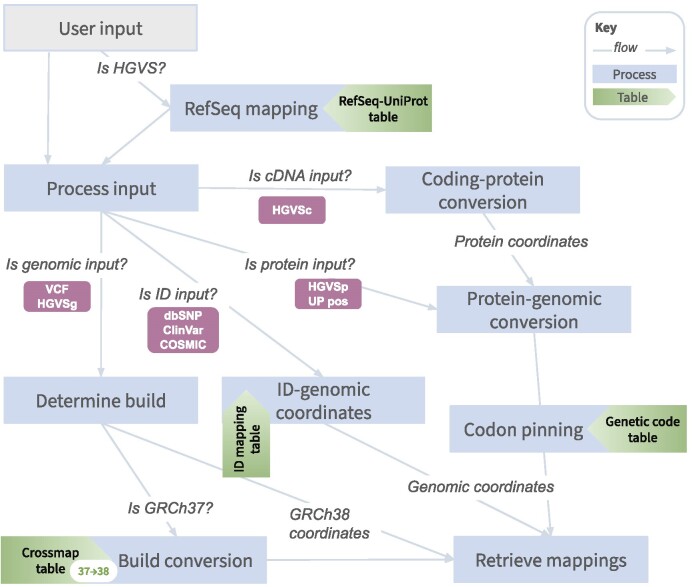
Schematic to show how ProtVar Maps different variant input types to protein and genomic position using pre-constructed tables.

Input format is assessed for type and then all input types are mapped to GRCh38 coordinates and then UniProt (18) canonical isoform positions for retrieval of annotations as shown in Figure 1. An equivalency table between GRCh37 and GRCh38 containing 37 025 561 positions was built using CrossMap (19). ProtVar compares the reference alleles given by the user (when more than 10 variants are entered) with those at the corresponding positions in the two assemblies to ascertain which build the data is likely from. The table is also used to convert GRCh37 to GRCh38 coordinates. A RefSeq-UniProt table was built to contain mappings between RefSeq (20) sequence IDs and UniProt accessions for 105 251 RefSeq IDs and was used in the mapping of HGVSc, HGVSg and HGVSp inputs. For cDNA inputs the RefSeq ID is mapped to the corresponding UniProt accession and then the protein position is derived from the coding position.

For protein inputs the codon sequence is retrieved from pre-mapped nucleotide-UniProt position tables. The variant amino acid is then used with the codon table to retrieve the nucleotide(s) which could cause the missense variation. ID inputs are mapped to genomic positions via pre-mapped tables derived from dbSNP (21) (950 139 431 IDs), COSMIC (22) (8 502 081 IDs) and ClinVar (23) (4 781 976 IDs). The reference GRCh38 assembly nucleotides and UniProt canonical isoform amino acids are used for the output even when they do not match the user entered reference. Details on pre-calculated tables can be found in supplementary methods.

### Functional data annotations

Functional annotations are retrieved using UniProt Proteins API (24) once per UniProt update cycle and cached for rapid retrieval by ProtVar UI and API. Features for the variant position are retrieved as well as position ranges which include the variant position, all with linked primary source citations. Nucleotide level pathogenicity predictions were retrieved from CADD v1.6 (25), amino acid level predictions were retrieved for the 3219 available proteins from EVE (26) and for all canonical sequence positions from ESM-1b (27). Amino acid conservation values were retrieved from VarSite which uses alignments with sequences from UniRef90, scored using the ScoreCons algorithm (28).

ProtVar pre-caches all human missense variants (including synonymous and stop gain) from UniProt variants DB fetched via Proteins API. This includes data from Ensembl (29), COSMIC, ClinVar, GnomAD (30) and other sources. In total ProtVar has 28 317 272 missense variant records representing 9 154 898 different variants in 6 157 589 protein locations.

Human structures from the AlphaFold Protein Structure Database (31) were used to predict protein stability and pockets. The structures were cleaned by removing low-confidence regions. These regions were defined as having an average pLDDT below 50 over a 10 amino acid window. FoldX v5.0 stability predictions (32) were then performed using the BuildModel command for all possible mutations at all positions in the structure. FoldX was chosen among alternatives as it is an unsupervised method, circumventing the possibility of overfitting to stability training datasets. Over 200M FoldX stability changes are precomputed in ProtVar. Pockets were predicted with AutoSite (33) using default parameters. Pocket predictions were then further filtered by mean pLDDT of pocket-associated residues to remove spurious pockets forming in regions of high uncertainty (34). Alphafold-Multimer (35) was used to predict the structures of binary protein-protein interactions. The standard sequence databases and tools to generate the multiple sequence alignments were used with default values for all parameters. Structures were post-processed using the FoldX RepairPDB command.

PDBe Mol* (36) is used to display all protein structures. Structures are retrieved from the PDBe via the API and the most recent AlphaFold2 structures are retrieved from the AlphaFold API. The predicted align error graph is taken from AlphaFold. Predicted pockets are highlighted on the same AlphaFold structure as the pocket prediction was calculated upon. The models displayed in the protein-protein interface view have been trimmed so only positions with a pLDDT over 70 are shown, as was the cut-off used in the interaction calculations.

### ProtVar implementation

ProtVar comprises a web-based user-facing service, a backend API responsible for delivering data to the website, and several integrated importers within the data production pipelines. The website operates as a Single Page Application (SPA) developed using React + TypeScript, dynamically fetching content from the API as users interact with the application. Notably, it utilizes the PDBe-molstar library [https://github.com/molstar/pdbe-molstar] for structure visualization, including features such as pockets and interfaces.

On the backend, the primary API is developed in Java using the Spring Boot framework, supported by RabbitMQ and Redis servers for request handling and caching, respectively. All components are containerized and deployed on a Kubernetes cluster, ensuring portability across various cloud service providers. The API is publicly exposed and documented using the OpenAPI specification, enabling programmable access to ProtVar data for integration into user pipelines.

ProtVar relies on a PostgreSQL database to store precomputed mappings, scores, predictions, and more. Genomic-to-protein mapping is calculated using UniProt, Uniparc (37) and Ensembl as primary data sources, aiming to align with their release cycles. Furthermore, this mapping is enriched with annotations obtained from Proteins, Variation API, PDBe API (38) and AlphaFold. The ProtVar API code can be found here: https://github.com/ebi-uniprot/protvar-be and the user interface code can be found here: https://github.com/ebi-uniprot/protvar-fe. Licensing and privacy statements, including the use of Google Analytics can be found in the ‘About’ section at the top of each page in ProtVar.

## Results

### ProtVar web server

**Figure 2.**
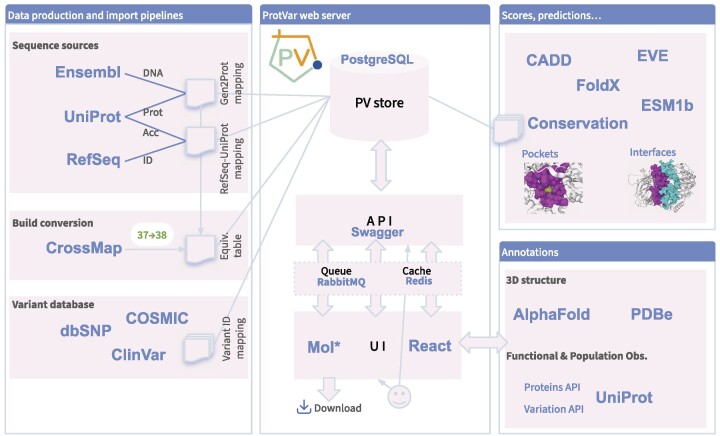
Resources used by Protvar to map and contextualize human missense variation. Most of the mappings and annotations are stored in tables or pre-cached with each update to assist rapid retrieval of data.

ProtVar annotates lists of human missense variants with functional and structural annotations in order to facilitate their contextualization and interpretation. It contains annotations and structures for 19 038 (over 93%) of the SwissProt (39) curated proteins from numerous sources as shown in Figure 2. ProtVar can process genomic, cDNA and protein inputs as well as several commonly used IDs. A comprehensive ‘help’ section is available from the top of all pages in ProtVar to guide users through functionality and all of the biological fields.

ProtVar shows a standardized mapping of variants to their position in proteins, even given mixed input types and returns them almost instantaneously. Three categories of annotations are provided to the user to contextualize the effect of variation in each specific case; Functional annotations, population observations and structural annotations. Functional annotations outline the reference function of the amino acid position, region and protein to help the user to understand the severity and potential for altering the position. Population observations highlight any diseases previously associated with the variant and also other variants reported at the same amino acid position to give clinical context. ProtVar also maps each user variant to every experimental structure possible and to the most recent AlphaFold2 model in addition to showing the relative positions of protein pockets and interfaces to the variant.

### Mapping variants to proteins

**Figure 3.**
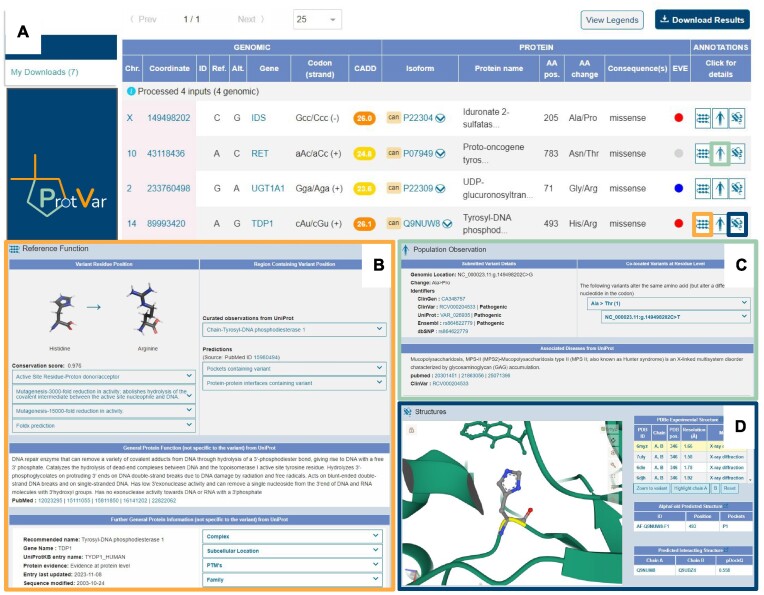
Annotations retrieved for four example variants. (**A**) Mapping table showing the annotations used for functional annotations (orange) population observations (green) and structures (blue). (**B**) Functional annotations for TDP1/Q9NUW8 H493R, (**C**) co-located variants at the same amino acid as RET/P07949 N783T. (**D**) Variant TDP1/Q9NUW8 H493R highlighted on an experimental structure.

ProtVar can process variant inputs in VCF, HGVSg or custom formats of any size either pasted or uploaded. It can also process cDNA position changes in HGVS format and automatically detect which genome assembly the variants are from. ProtVar can also handle variants as amino acid position in proteins as HGVSp or using the UniProt accession and position as well as processing ClinVar, COSMIC and dbSNP IDs. Examples of each of the formats can be found for rapid testing as buttons to the right of the paste box. A full list of all the formats supported can be found in Supplementary Table S2. The mapping table serves as the results home page from where users can further explore annotations for each variant via links and the three annotation buttons at the end of each row, an example is shown in Figure 3A using the mixed format ‘genomic’ examples from the search page.

ProtVar shows the protein position in the UniProt canonical isoform by default, but clicking on the arrow by the accession users can explore the variant position in other isoforms. The mapping table also shows users the codon change, the CADD pathogenicity prediction based on the nucleotide change and the EVE pathogenicity prediction based on the amino acid change. Any issues with inputs or transformations of the data by ProtVar, for example changing from GRCh37 to GRCh38 assembly coordinates, are also displayed in the table.

### Annotations: reference function

By clicking on the functional annotation button towards the end of each row users can explore all current knowledge on the position and region collected from literature by UniProt and other sources. In the example in Figure 3B for TDP1 the left panel shows the annotations specific to the amino acid position. Here we can see that the amino acid conservation for the position is very high (0.976) and we can see that it has been flagged as an ‘active site residue - proton donor/acceptor’. ProtVar also shows us that two mutagenesis experiments have demonstrated that changing the amino acid at this position results in a reduction in activity with links to the primary evidence included. Finally, ProtVar shows us the predicted change in the protein free energy after mutating the protein at this position from histidine to Arginine. Moving to the annotations in the RET protein we can see that while there is little known about the position specifically, the right panel shows that the region containing the variant is in the cytoplasmic part of a transmembrane domain and that the domain is functional in the protein kinase family. Additionally, mutagenesis in the region causes loss of induced cell death but increased cell aggregation. The functional annotation screen also shows general protein information, not specific to the variant position or region but potentially important to understand the consequence of the variant on the organism including a referenced description of protein function from literature, names, synonyms and information about when the sequence and functional data was last updated.

Finally, functional information form UniProt including catalytic activity, partners in protein complexes, cellular location, PTMs and protein family are included along with links to their evidence sources and data concerning the canonical isoform including the versioned Ensembl gene, protein and transcript ID(s)

### Annotations: population observations

The Population Observations section shows information regarding the entered variant in other resources. In the example for RET in Figure 3C ProtVar shows us that the entered variant has also been reported in ClinGen (40), ClinVar, Exac (41), TOPMed (42), dbSNP and gnomAD. It has been classified as likely benign by ClinVar and each ID is linked to the entry in the corresponding resource. The information below the left panel show the diseases associated with the variant from literature which are retrieved from UniProt. ProtVar retrieves variants from a large pool of other resources which are co-located at the same amino position in the protein, not exclusively those variants which are genomically colocated. The panel to the right shows that the same amino acid position also has other variants associated with it. An A/G nucleotide change in the same coordinate position results in an Aspartate variant, reported in ClinGen and gnomAD. An A/G nucleotide change in the third position of the variant codon results in a change to Serine. Whilst this is a different coordinate position, it affects the same amino acid and has the same disease associated with the variant, however there is also an additional disease associated, Hirschsprung disease 1.

### Annotation: structure

**Figure 4.**
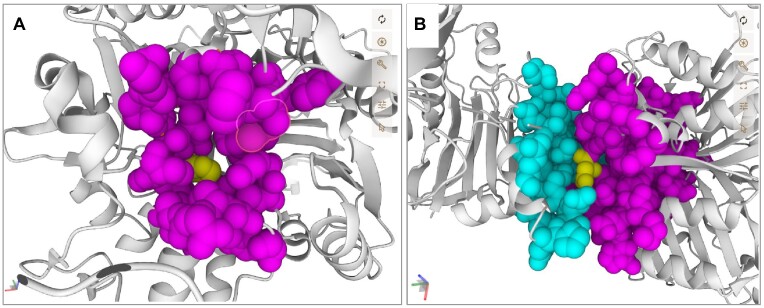
Variants involved in predicted structural regions. (**A**) Variant at position TDP1/Q9NUW8 H493R predicted to be in a pocket. (**B**) Variant at position TDP1/Q9NUW8 D256H predicted to be involved in an interface with APEX2/Q9UBZ4.

ProtVar provides mapping to experimental and predicted structures with visualizations to show variant location as shown in Figure 3D. Also the position of variants in relation to predicted pockets and interfaces between protein-protein interactions. In the example for TDP1 there are 42 experimental structures which contain position 493. Users can choose which structure to observe the variant position in, zoom to the position and select specific chains using the buttons below the table. Although some proteins do not have experimental structures, ProtVar does show every variant on the latest AlphaFold2 model in the table below the experimental structure table. The structures are coloured by pLDDT confidence from AlphaFold and the pairwise align distance graphic is also shown for each structure. If a protein pocket is predicted, as is the case in the example for TDP1, users can observe the pocket using the button below the table (as shown in Figure 4A) which colours and highlights the pocket and hides pLDDT colouring.

The final structure table shows the predicted protein-protein interaction models where the variant is predicted to be near the interface, ordered by the pDockQ score representing model confidence. The interface residues at 8 Å inter chain distance can be highlighted by clicking the ‘highlight interface’ button. Figure 4B shows the TDP1 example but with position D256H highlighted.

### Data retrieval

In addition to online viewing, ProtVar offers users the functionality to download mappings, either with or without annotations, facilitating offline analysis or processing. These downloaded files can be easily managed within the dedicated ‘My Downloads’ section of the web resource. Furthermore, the REST API provides programmatic access, allowing users to automate data retrieval and integrate it into any downstream pipelines with ease. The dedicated API documentation page provides users with example requests and the corresponding expected response structures. This interactive feature enables users to directly engage with the API within the page interface.

## Discussion and future direction

The increasing interest in the interpretation of missense variation, the increase in available variants and in annotations mean that tools need to adapt and improve to meet changing challenges and needs. ProtVar aims to represent the most flexible and user-friendly resource currently available for the interpretation of human missense variation. With over 93% of human curated proteins, as well as alternative isoforms and transcripts, and structure for every human protein pre-mapped, researchers are more likely to rapidly find annotations for their variant list (of any size) in ProtVar than in other currently available resources. The flexibility of input also means that in addition to those with genomic coordinates, researchers with cDNA inputs, protein positions or IDs can retrieve annotations without further formatting, as can investigators who are not sure which assembly their variants are from. Any white spaces, delimiters and mixed formats are interpreted whenever possible meaning that more input varieties than anywhere else can be run and homogenized mappings can be retrieved.

Annotations in ProtVar are predominantly focussed at the amino acid position level and the variation type is limited to human missense variation. This allows ProtVar to leverage the wealth of annotations from UniProt and seamlessly map to AlphaFold structures which mirror UniProt position numbering. ProtVar also uniquely provides co-located variation at the amino acid level rather than the nucleotide level reflecting variants which may affect the same protein function rather than those that strictly share the same mutation location. ProtVar also brings stabilization, pocket and interface predictions alongside curated annotations to facilitate molecular consequence interpretation of missense variants. We hope that the ability to retrieve specific types of annotations via the tailored download option and the programmatic access will give users the required flexibility to facilitate the interpretation of their variants. Indeed several resources already link to ProtVar to help their users to interpret missense variation. UniProt, for example, redirects users to the position specific annotations in ProtVar from its variants page. Open Targets directs users from its Genetics Platform (43) to ProtVar to facilitate the interpretation of variation in terms of drug target assessment.

ProtVar does not replace complementary resources, instead focusing on SNVs in human coding regions. VEP for example should be used for non coding regions and Missense3D should be used for an in depth analysis of structural changes for single positions. However we believe that ProtVar does represent a useful addition to the variant analysis field with its speed, breadth and annotation, prediction types and programmatic access, which will be useful to a diverse spectrum of the research community. Along with regular mapping and annotation updates coupled with the UniProt update cycle we will further develop the visualizations, including enhancement to model confidence for pockets and interfaces. We will continually expand coverage with the aim of mapping to all human proteins as well as all known transcripts and isoforms. We also aim to expand our variant consequence reporting and variant types supported to include small indels.

## Supplementary Material

gkae413_Supplemental_File

## Data Availability

The data underlying this article are available from https://www.ebi.ac.uk/ProtVar. The ProtVar API code can be found here: https://doi.org/10.5281/zenodo.11103699 DOI: 10.5281/zenodo.11103699 and the user interface code can be found here: https://doi.org/10.5281/zenodo.11103695 DOI: 10.5281/zenodo.11103695.
