# Supplementary material for: ProtVar: mapping and contextualizing human missense variation
Source: Nucleic Acids Res. 2024 May 20;52(W1):W140–7. doi: 10.1093/nar/gkae413 (PMC11223857; doi:10.1093/nar/gkae413)
Supplement: gkae413_Supplemental_File [file gkae413_supplemental_file.pdf]

SUPPLEMENTARY DATA

	ProtVar	VEP	VarMap/VarSite	MisCast	MutFunc	Phyre Risk
**Input types**						
Protein position	Y	N	N	Na	Y	Y
ID types	3	1	N	Na	N	1
cDNA	Y	Y	N	Na	N	Y
Build detection	Y	N	Y	Na	N	N
Species	1	27	1	1	3	1
Variant types	SNV	all	SNV	SNV	SNV	SNV+indels
**Accessibility**						
https	Y	Y	Y	N	N	N
Download	Y	Y	Y	Y	Y	N
API	Y	Y	N	N	N	N
Offline	N	Y	N	N	N	N
UI input->download time*	0:31	12:00	30:00	Na	1:30	No download
% proteome	93	94	94(VEP)	15	95	94(VEP)
Last updated	2024-03	2024-01	2019-03	2020	2018	2019-07
**Annotations**						
PTMs	Y	N	Y	Y	Y	N
Curated annotations	Y	N	Y	Y	Y	N
AA Colocated variants	Y	N	Y	N	N	N
AA Conservation	Y	N	Y	N	Y	N
Region function	Y	N	Y	Y	Y	N
AlphaFold	Y	Y	N	N	N	N
Protein-protein interfaces	Y	N	Y	N	Y	Via missense 3D-PPi
Predicted stability	Y	N	N	N	Y	N
Protein pockets	Y	N	N	N	N	Via Missense 3D

Supplementary table 1. Comparison of ProtVar with tools with overlapping functionality. The fields are from a protein missense variant point of view specifically. Many tools are not focussed on this and have other important information.*random 1000 ClinVar missense variants, from data input to download ready.

Input type	Description	Format	Params	Examples	(Pre)processing
Gen	Genomic input	VCF, HGVSg, gnomAD	(id), chr (or NC accession), pos, (ref), (alt)	X 149498202 . C G<br>NC_000010.11:g.43118436A>C<br>1-55505447-C-T	Genome assembly detection and conversion if required
cDNA	Coding DNA	HGVSc	refSeqID,cDNApos, ref, alt	NM_000202.8:c.1327C>T<br>NM_020975.6(RET):c.3105G>A (p.Glu1035Glu)<br>NM_000463.3(IDS):c.1124C>T	Coding-to-protein position conversion and RefSeq ID (NM) mapping
Pro	Protein input	HGVSp, Uniprot acc, pos	acc, pos, (ref), (alt)	P22304 A205P<br>P07949 asn783thr<br>NP_001305738.1:p.Pro267Ser	Protein-to-genomic coordinates mapping and RefSeq ID (NP) mapping
ID	Variant ID	dbSNP, ClinVar, COSMIC	id	RCV001270034<br>COSM1667583<br>rs864622779	Genomic coordinates lookup for ID

Supplementary table 2. The formats and descriptions of the variant inputs which can be processed by Protvar. Fields in brackets are optional.
